# Recent Advances in Treatment of Coronary Artery Disease: Role of Science and Technology

**DOI:** 10.3390/ijms19020424

**Published:** 2018-01-31

**Authors:** Eswar Kandaswamy, Li Zuo

**Affiliations:** Radiologic Sciences and Respiratory Therapy Division, School of Health and Rehabilitation Sciences, The Ohio State University College of Medicine, Columbus, OH 43210, USA; eswark1211@gmail.com

**Keywords:** stem cells, surgery, treatment modality, heart

## Abstract

Coronary artery disease (CAD) is one of the most common causes of death worldwide. In the last decade, significant advancements in CAD treatment have been made. The existing treatment is medical, surgical or a combination of both depending on the extent, severity and clinical presentation of CAD. The collaboration between different science disciplines such as biotechnology and tissue engineering has led to the development of novel therapeutic strategies such as stem cells, nanotechnology, robotic surgery and other advancements (3-D printing and drugs). These treatment modalities show promising effects in managing CAD and associated conditions. Research on stem cells focuses on studying the potential for cardiac regeneration, while nanotechnology research investigates nano-drug delivery and percutaneous coronary interventions including stent modifications and coatings. This article aims to provide an update on the literature (in vitro, translational, animal and clinical) related to these novel strategies and to elucidate the rationale behind their potential treatment of CAD. Through the extensive and continued efforts of researchers and clinicians worldwide, these novel strategies hold the promise to be effective alternatives to existing treatment modalities.

## 1. Introduction

The scientific advancements in the understanding of the pathophysiology of coronary artery disease (CAD) have led to a decrease in the mortality (in age-adjusted subjects) towards the turn of the 20th century [1]. However, CAD remains one of the leading cause of death in the world [2]. CAD is responsible for one-third of deaths in developing and developed countries in people over 35 years of age, with the percentage reaching close to 50% (according to some estimates) in western countries [3,4]. The worldwide burden is set to reach 47 million disability adjusted life years (years lost due to disability, ill-health or death) by the year 2020 as projected by World Health Organization [5]. In the United States alone, there are estimated to be 900,000 subjects who suffeedr or die from CAD and its complications in 2016 [6].

There has been a greater focus in research aimed at all aspects of CAD in the last decade. Due to exhaustive efforts from clinicians and researchers worldwide, there has been significant progress made in developing novel strategies for patients suffering from CAD and its associated complications. These strategies have ranged from drugs to robotic surgery to nanotechnology. This article will summarize the literature on the recent advances in coronary artery disease research in respect to therapeutics and biomarkers. This article will cover topics under the following headings: robotic surgery, nanotechnology, stem cells and other related advancements.

## 2. Robotics

**Figure 1:**
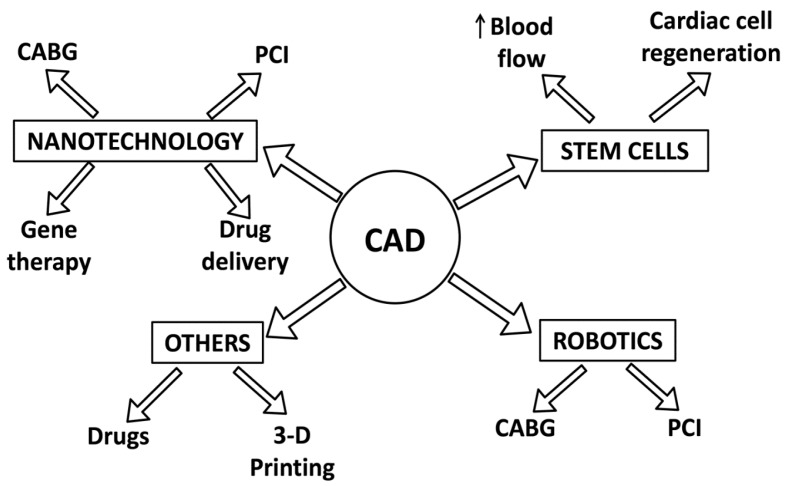
This schematic illustrates the potential applications by which nanotechnology, stem cells, robotics, new drugs and 3-D printing can be used in the treatment of coronary artery disease. Abbreviations: coronary artery bypass graft (CABG), coronary artery disease (CAD), percutaneous coronary intervention (PCI). The upward arrow represents an increase of blood flow.

**Figure 2:**
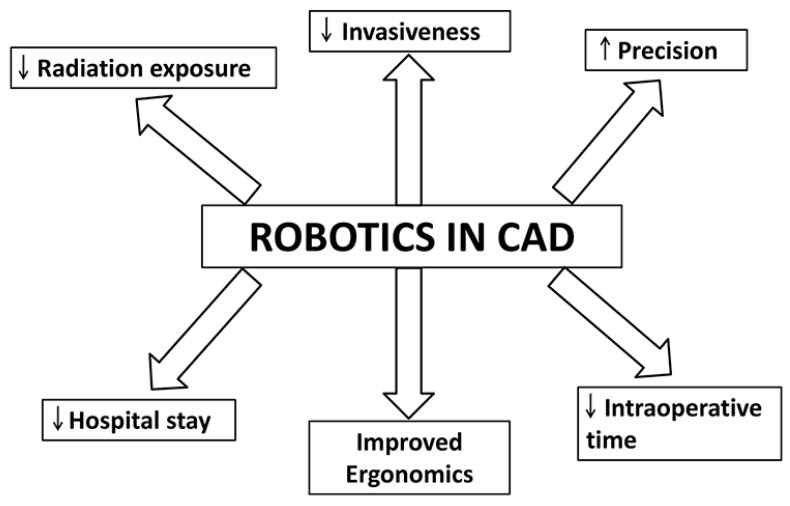
The schematic illustrates the potential advantages of using robotics in the treatment of CAD. The upward or downward arrows represent an increase or a decrease of blood flow, respectively.

Robots have been in place in mass production industries for many years. However, their introduction in medicine was fairly recent and started in the fields of surgery and radiotherapy. In cardiology, they have been in use for more than a decade for surgeries like mitral valve repair, coronary artery bypass graft and septal defect closure. The technology is fast evolving with reports emerging about their potential applications in percutaneous coronary intervention (Figure 1) and atrial fibrillation ablation [7]. Robotics provide the operator with advantages such as improved ergonomics, precision and sometimes shortening of intraoperative time (Figure 2) [7]. There have been reports that robot-assisted surgery can shorten patient hospital stay and improve patient perception (Figure 2) [8].

In the field of interventional cardiology, robotics are being used for catheter-based surgical procedures. Conventional angiography radiation exposure for CAD patients is estimated at 7 mSV, and this exposure can be increased by up to 5 times in complicated surgeries [9]. Robotic guided surgery has potential to limit this radiation exposure. In addition, they can also reduce contrast induced nephrotoxicity and associated mortality in patients (Figure 2) [9]. In terms of patient related outcomes, the robotic assisted surgery has potential benefits as it can accurately measure the size of the lesions (which can be miscalculated using 2D angiography) which could improve long-term health. Hence, they reduce radiation exposure for the surgeon and the patient as well as improve precision by rendering accurate measurements of lesions (Figure 2) [7]. Granada et al. published the first robotic interventions for cardiac patients [10]. They performed coronary angioplasty and reported 100% success rate (measured in terms of less than 30% residual stenosis along with the absence of major cardiac complications) in all of their patients (80 subjects) [10]. In a multicenter study published by Weisz et al., a percutaneous coronary intervention was performed for patients with coronary artery disease [11]. They used similar success criteria (measured in terms of less than 30% residual stenosis along with absence of major cardiac complications) and reported a 97.6% rate of success (164 patients) [11]. They also reported a significant reduction (95%) in operator radiation exposure [11]. 

Robotics has also been used to perform coronary artery bypass grafting in CAD patients (Figure 1). The procedure, including the harvesting of the mammary arteries and anastomosis, can be performed endoscopically [7]. The results of the clinical studies are summarized in Table 1. Although there are reported benefits for robotically assisted bypass grafting, high costs and long learning curves have slowed down its progress towards becoming used routinely [12]. Robotically assisted hybrid coronary revascularization, which involves coronary artery bypass graft as well as percutaneous coronary intervention, has also been developed as a treatment modality for CAD. There have been reported benefits such as reduced morbidity and shortened hospital stays due to the minimally invasive nature of the procedure [13,14,15,16].

The current state of robotic surgery is promising in the treatment of CAD. These systems are of excellent quality with high-end technology. Their proposed benefits in the form of improved precision, increased visibility, improved ergonomics and reduced radiation exposure have been documented, which have translated into improved patient recovery times with reduced hospital stays [7,20,21]. They also provide a distinct advantage for procedures that are difficult to be performed using endoscopy or catheters [7]. However, their translation into full-fledged clinical usage is inhibited by high cost and the learning curve needed to master these procedures [22]. It remains to be determined, with further technological advancement, whether this technology will be accepted into routine clinical practice and replace conventional technologies.

## 3. Nanotechnology

Nanotechnology has been revolutionizing several fields including medicine. It involves the engineering of nanoscale molecules with distinctly different properties than bulk molecules of the same composition. These inherent differences provide distinct benefits which are strong reasons for the boom in nanotechnology research. This technology has been studied in CAD for its potential benefits in medical (non-invasive) and invasive treatment modalities, drug delivery applications, percutaneous coronary interventions, gene therapy, and coronary artery bypass graft (Figure 1).

**Figure 3:**
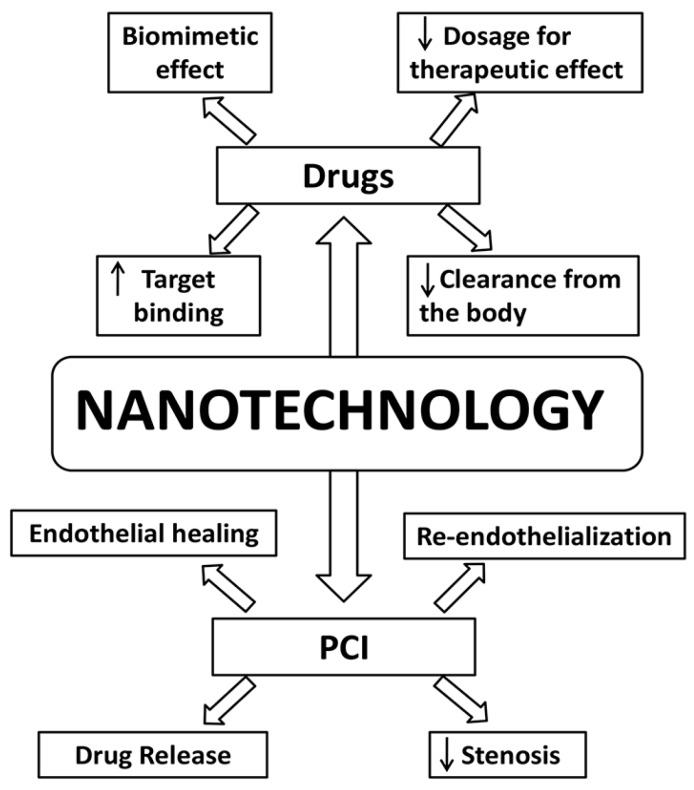
The schematic illustrates the potential advantages of using nanotechnology in the treatment of CAD. PCI—Percutaneous coronary intervention. Abbreviations: percutaneous coronary intervention (PCI). The upward or downward arrows represent an increase or a decrease of the referred parameters.

Cholesterol is an important factor involved in the pathogenesis of coronary artery disease. High levels of low-density lipoproteins (LDL) are implicated in coronary artery disease whereas high-density lipoproteins (HDL) are thought to have a protective role since they are involved in transportation of cholesterol away from the peripheral tissues. Nanotechnology has been used in the synthesis of a dimyristoyl phosphatidylcholine, which mimics the surface characteristics of HDL (Figure 3) by mediating the removal of cholesterol from the peripheral tissues and transport it to the liver. In an animal model study, mice fed with a cholesterol-rich diet showed significantly lower plaque volume and cholesterol content in the aorta when treated with dimyristoyl phosphatidylcholine liposomes [23]. Fumagillin is an anti-angiogenic drug that has been shown to inhibit angiogenesis thereby promoting plaque regression in coronary arteries. One of the disadvantages that has prevented Fumagillin application is its ability to cause adverse neurocognitive effects at high doses, which is required to achieve a therapeutic effect. Winter et al. demonstrated that the Fumagillin can be delivered through αvβ3 integrin targeted nano-delivery system, and is able to achieve significant antiplaque effects at one-third of the usual dose (Figure 3) [24]. Several nanoparticle-based antithrombotic agents have been tested for their potency. d-phenylalanyl-l-prolyl-Larginyl-chloromethyl ketone is a potent antithrombotic agent that is rapidly cleared from the body, thus limiting its clinical use [25]. When combined with a perfluorocarbon-core nanoparticle, it has been shown to have improved antithrombotic action, as shown by Myerson et al. in an animal model study (Figure 3) [26]. Peters et al. on the other hand used hirudin with fibrin binding micellar nanoparticles which exhibited greater targeting of fibrin clots in vivo (Figure 3) [27]. Collagen IV nanoparticles have been tried in an animal model study and were shown to improve collagen formation while reducing oxidative stress by mimicking Annexin A1 (glucocorticoid regulatory protein) [28]. 

The studied modifications were in the form of liposomal glucocorticoid carrier (to deliver anti-inflammatory hormones thereby reducing arterial wall inflammation), lipid nanoparticles (to deliver siRNA antagonistic to the C-C chemokine type 2 pro-inflammatory receptor), and HDL nanoparticles (to deliver simvastatin to inhibit monocyte recruitments) [29,30,31]. Gel-based nanoparticles combined with rapamycin (antiproliferative and antiapoptotic effect) were studied in an animal model, which were found to re-endothelialize injured arteries and reduce hyperplasia [32]. Smart nanoparticles such as a pH-dependent delivery of antioxidants as developed by Tang et al. has shown promise in treating cardiac diseases [33].

Nanotechnology has shown potential benefits when used in percutaneous coronary intervention. They have been studied for their ability to release drugs as well as promote healing and reduce restenosis (Figure 3) [25]. Nano-sized hydroxyapatite coating for controlled release of sirolimus (an immunosuppressive drug) performed satisfactorily in clinical trials [34]. Similarly, the release of sirolimus was studied using carbon nanoparticle coated stents with consistent drug release, as reported in an in-vitro study [35]. The sirolimus-releasing stents were compared with pitavastatin nanoparticle-eluting stents. The latter were found to be more efficient in terms of faster endothelial healing while being comparable in other parameters (Figure 3) [36]. Magnetic silica nanoparticles were loaded with rapamycin, coated onto the stent and exhibited rapid endothelialization in in vivo studies [37]. Endothelial healing and re-endothelialization help to restore the injured vessel back to health. Polycaprolactone was found to be an effective carrier for nitric oxide to prevent restenosis (Figure 3) [38]. In animal model studies, it has been proven that liposome encapsulated alendronate (a bisphosphonate) can reduce restenosis and neointimal formations (Figure 3) [39]. Similarly, paclitaxel (antimitotic drug) in the form of albumin-based nanoparticles have shown to have significant antiproliferative and restenosis effects without significant toxicity even when administered systemically [40,41]. The nanoparticles in these cases were either used to improve cell membrane permeability (alendronate) or binding capacity to the targeted tissues (paclitaxel) [42]. Polymeric stent coatings in the form of poly(lactic-*co*-glycolic acid) were proven to have a controlled release of the drug paclitaxel (nanocoatings-64) and polyethylene glycol was proven to reduce platelet adhesion [38,43]. Nanomodifications have also helped scientists in targeting specific delivery of medications such as collagen IV, chondroitin sulfate, tissue factor, or stents [44,45,46,47].

Nanotechnology has the potential to promote healing by inducing endothelialization of the stent (Figure 3) [25]. These nano-modifications have been in the form of nanofibrous matrix (attracts endothelial cells), polyhedral oligomericsilsesquioxanepoly-(carbonate-urea) urethane (improved adherence and proliferation of human endothelial cells), peptide amphiphile-nanofiber coating (for promotion of endothelial cell adhesion), and magnetic nanoparticles (for preferential movement of cells towards the stent) [48,49,50,51]. Nanotechnology also has potential applications in finding synthetic alternatives for coronary artery bypass grafts. Researchers have studied the potential of electrospunnanosized fibrous scaffolds, which may prove to be an alternative synthetic graft for coronary artery bypass graft procedures [52,53]. Targeting drug-eluting stents in gene therapy is another area where nanotechnology holds promise. Gene eluting stents can be used to overcome restenosis, in-stent thrombosis, and delayed endothelialisation [54,55]. Several nano-coatings in the form of hyaluronic acid (to carry pDNA), nanobiohybrid hydrogel (to carry Tat peptide and DNA), and poly(lactic-*co*-glycolic acid) nanoparticles (carrying PDGF receptor-β antisense RNA) have been studied in animal models and have shown promising results [56,57,58]. Other gene targets that have been studied extensively include antisense oligonucleotide, chitosan-plasmid DNA, Akt1 siRNA, vascular endothelial growth factor, prostacyclin synthase, and endothelial nitric oxide synthase [54]. 

Nanotechnology has led to an interesting and promising direction in the treatment of CAD. It has valuable potential in delivering drugs that are otherwise limited by their pharmacokinetics. Its applications in stent and gene therapy are potentially useful for future therapeutics based on these modalities. Further randomized controlled trials need to be conducted to establish strong evidence to support the use of these newer technologies for CAD treatments. This needs to be carried out with strong collaboration between researchers, engineers, biomedical engineers, nanotechnologists and clinicians. As the technology and evidence develops, we will soon enter an era where existing established treatment modalities could be questioned and eventually replaced by nanotherapeutics.

## 4. Stem Cells

**Figure 4:**
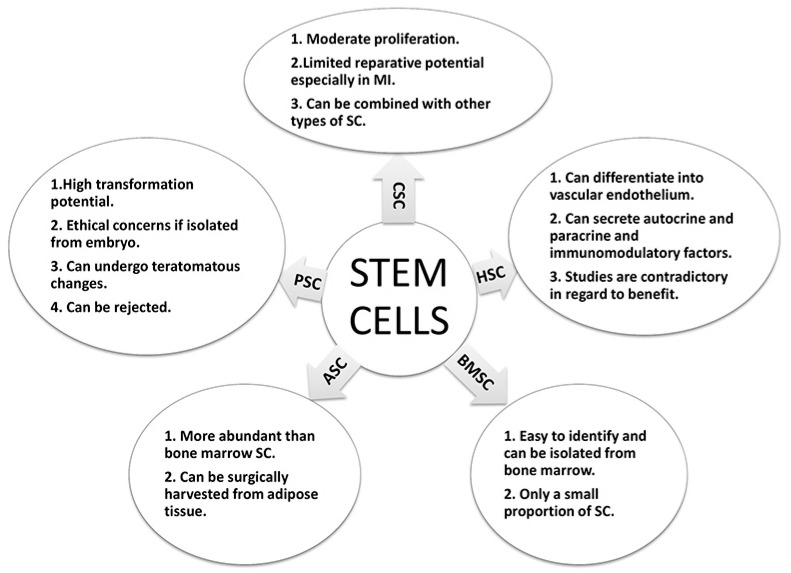
The schematic illustrates the potential of different types of stem cells in the treatment of CAD. Abbreviations: Adipose stem cells (ASC); bone-marrow stem cells (BMSC); cardiac stem cells (CSC); hematopoietic stem cells (HSC); pluripotent stem cells (PSC); stem cells (SC).

Research in cardiovascular disease has sought to repair myocardial damage and increase blood supply in ischemic conditions of the heart, thereby reversing the effects of CAD. In this respect, both vascular growth factors and stem cells have generated a lot of interest as a mode of treatment in patients with CAD [59]. The rationale behind such therapy is to improve the blood supply to ischemic areas of the heart by stem cells, as well as promote cardiac cell regeneration (Figure 1). This can be achieved in one of two ways: by a direct effect of the stem cells, or by paracrine factors secreted by these stem cells [60]. In this regard, hematopoietic stem cells have been of great interest, especially for mononuclear cells and endothelial progenitor cells (Figure 4). Studies conducted using these cells for various forms of ischemic heart disease (such as acute myocardial infarction (MI) and chronic ischemic heart disease) have been contradictory, although some studies have demonstrated a beneficial effect in such patients [61,62,63,64]. This has led to the inclusion of other types of stem cells, such as adipose derived stem cells, into such studies. A novel alternative is the creation of induced pluripotent stem cells, for which adult cells are transformed into pluripotent stem cells, similar to embryonic stem cells [65,66]. Although it offers a promising alternative, concerns of cancerous transformation of the undifferentiated stem cells have to be taken into account before they can be tried in human subjects.

The stem cells studied in cardiovascular research ranged from bone marrow to adipose tissue to skeletal muscle stem cells. Bone marrow-derived mononuclear cells are the most readily available cells for transplantation in the body. They are easy to identify based on their cell surface markers and can be isolated from the bone marrow [60]. However, their therapeutic potential is low since the harvested cells contain a multitude of cells with a small proportion of stem cells (Figure 4) [63,67]. The bone marrow-derived mesenchymal stem cells are found in even lower concentrations than that of mononuclear cells thus requiring several weeks of maturation with different growth factors in the lab prior to clinical usage. The adipose derived stem cells can be surgically harvested from adipose tissues. They are more abundant in comparison to the bone marrow-derived cells. This drastically reduces the time and cost involved in laboratory procedures to culture them for clinical use (Figure 4) [68]. The pluripotent stem cells have a high potential for transformation. Although embryos represent the most obvious source of stem cells, their use has ethical concerns and is in debate. Additionally, these cells could potentially face rejection when transplanted to a recipient. However, it is possible to reprogram adult cells and transform them into pluripotent cells (similar properties as embryonic stem cells), thereby being called induced pluripotent stem cells. These cells can be auto-transplanted and therefore would not be rejected. However, due to their transformation potential, unless closely regulated, they can undergo teratomatous (derived from all three germ layers) changes in the body (Figure 4) [65,69]. Due to the risk of teratomatous changes, this area of research requires more work before they can be considered safe for human trials. Another interesting source of stem cells are cardiac stem cells. Although the heart was considered as a static organ (with little or no potential to undergo mitosis during adulthood) [70,71,72], recent evidence has shown a different perspective. The heart is now believed to have intrinsic regenerative potential and undergoes constant turnover throughout adult life (Figure 4) [73]. Beltrami et al. showed that the heart possesses cardiac stem cells that could be responsible for the intrinsic regeneration and turnover throughout adult life [70]. These cells are more numerous in the apices of the atrium and ventricles [74]. Although these cells are known to be involved in tissue homeostasis, their reparative potential is limited, especially in conditions with extensive damage such as myocardial infarction (MI) [72,75,76,77]. More recently, there has been an interest to develop and inject multiple stem cells that can communicate with each other, termed as a “Cardiocluster”. These clusters are cocktails of cells that include cardiac progenitor cells, mesenchymal stem cells, endothelial progenitor cells and fibroblasts (Figure 4). They have the potential to promote cardiac cell regeneration in disease states where cell function is reduced such as CAD [78]. 

The clinical data for stem cell therapy is in its early days with reported literature covering both non-randomized and randomized trials. One non-randomized trial reported improved left ventricular ejection fraction (LVEF) function following injection of mononuclear stem cells in patients with MI within three months [79]. Improved exercise capacity, reduced mortality and scar tissue are shown in a 5-year follow up [80]. Several other studies showed similar effects following treatments with mononuclear stem cells after MI [64,81,82,83]. An earlier meta-analysis reported an improvement in LVEF function by 2.99% following bone marrow stem cell transplantation in patients after MI [84]. However, the meta-analysis did not include recent studies that reported no improvement in left ventricular function [61,62]. In patients suffering from chronic ischemic heart disease, there is reported evidence towards improved cardiac function following the use of bone marrow derived-stem cells [85,86,87,88]. There have been several trials that have studied the clinical efficacy of mesenchymal stem cells. They have reported an improvement in cardiac function and relative safety in the use of mesenchymal stem cells [89,90,91,92]. Cardiac derived stem cells have also undergone clinical testing and have shown promising results [93,94,95]. They reported an improvement in LVEF [93], an improvement in the left ventricular mass that was viable [94], improved quality of life [93], reduced scar mass, improved regional contractility [95] and safety of the procedure [93,94]. Interestingly, a patient that was treated with cardiac stem cells 14 months after MI had similar therapeutic benefit as someone treated earlier, suggesting that cardiac stem cells could be beneficial in chronic ischemia patients [96]. However, it should be noted that the observed clinical benefit was less than the expected clinical benefit based on prior in vitro and animal studies [97] (for a more in-depth review on this topic, the reader can refer to Kastrup [60], Quijada and Sussman [98] and Dixit and Katare [99]).

Stem cell therapy continues to be a promising treatment modality for coronary artery disease (both acute and chronic). The experimental and clinical studies have shown promising results. However, further research is needed to understand the exact mechanisms of action and the ideal source of stem cells to derive optimum benefit and to further our understanding. Several challenges have to be overcome (such as long term safety and route of administration), but the direction of current research looks promising.

## 5. Other Advancements

### 5.1. 3-D Printing

Cardiac conditions often require 3-D imaging such as magnetic resonance imaging, computerized tomography, and 3-D echography to diagnose and treat these conditions. The limitations to this are that though these images are in 3-D, they are viewed on a 2-D computer screen or films. Although it could be sufficient for some cardiac procedures, the current imaging modalities are not effective for more complex interventions [100,101]. 3-D printing has a potential role in CAD (Figure 1) as it cannot only overcome these limitations but also allow for complete visualization, tactile sense, education and surgical planning as well as simulation [102]. 3-D printing involves additive manufacturing of a model using 3-D data from imaging modalities. Scientists are starting to see the full potential of 3-D printing as the technology continues to evolve. In the field of cardiology it has tremendous potential in the treatment of congenital defects, cardiac tumors, cardiomyopathy, functional flow models, valvular heart diseases, stent placement for CAD and other cardiac surgeries [103,104,105,106]. 3-D printing allows the visualization of 3-D printed heart with coronary arteries in order to visualize the extent of occlusion and stenosis in CAD patients [107]. These models can be used in a pulsatile flow loop environment, not only to visualize and understand complex flow patterns but also to simulate interventions [105]. 3-D printed models are also useful in CAD research to compare imaging and treatment modalities [105,108]. One in vitro study mimicking a clinical scenario proved that 3-D printing could be more effective in planning and treating complex situations (bifurcation lesions) that require stent placement [106]. Tissue engineering models are now being tested to fabricate stem cells along with extracellular matrix (tissue printing) for implantation in the body [109]. In vitro studies have been successful in tissue printing cardiac cells in different scaffolds [109,110]. In animal model studies, the implantation of printed tissue in epicardial tissues showed beneficial effects including reduced adverse remodeling and improved perfusion in myocardial infarct models [109,110].

### 5.2. Drugs

CAD patients are often on supportive, therapeutic, and lifelong medication for the condition itself and co-morbidities (such as hypercholesterolemia). There have been recent advances in drug developments for CAD patients (Figure 1). One class of drugs taken by patients suffering from CAD are oral antithrombotic medications such as aspirin and clopidogrel. A few years ago a group of drugs collectively termed as novel oral anti-coagulants were discovered. This group consists of the following drugs: ximelagatran, darexaban, dabigatran, rivaroxaban, and apixaban [111]. Of which, dabigatran, edoxaban, rivaroxaban, apixaban are approved for clinical use. Dabigatran is a competitive inhibitor of thrombin while edoxaban, rivaroxaban, and apixaban are inhibitors of clotting factor Xa. Use of dabigatran in CAD patients was studied in a phase 2 trial. The results revealed that ischemic events in patients were significantly reduced at higher doses of the drug (110 and 150 mg), but this benefit was counteracted with a four-fold increase in bleeding risk. However, the trials concluded that lower dose therapy could be used without a significant increase in bleeding risk [112]. 

An important protein that controls the regulation of LDL is proprotein convertase subtilisin/kexin type 9 (PCSK9) [113,114,115]. They function to reduce the number of LDL receptors thereby decreasing LDL cholesterol levels in the blood [115]. Another important drug which could block the action of PCSK9 is Alirocumab. The drug itself is a monoclonal antibody produced by recombinant DNA technology [114]. The first studies reported a reduction in LDL cholesterol levels ranging from 28% to 65% depending on the route of administration (subcutaneous or intravenous) [116]. In phase II studies (randomized controlled double blinded trials) it was reported that LDL cholesterol reduction ranged from 18.2% to 67% (depending on the dosage) compared to placebo [116,117]. When combined with atorvastatin, Alirocumab brought about a LDL cholesterol reduction of 66–73% whereas placebo and atorvastatin brought about a reduction of 17% [118]. These results were confirmed in several phase III trials [119,120,121,122,123,124,125,126,127,128,129,130]. Since high LDL levels are linked to CAD, the use of Alirocumab reduced adverse cardiovascular events by 15–48% [127,131,132]. Another drug that was recently developed for the treatment of heart failure is the angiotensin receptor-neprilysin inhibitor (ARNi). This drug contains a combination of sacubitril and valsartan, commonly referred to as the LCZ696 or ARNi [133,134]. The valsartan portion is a drug of the angiotensin receptor blocker family as well as angiotensin II receptor antagonist, while the sacubitril component is neprilysin inhibitor [135]. This drug has proven to be more effective in the treatment of heart failure than traditional Angiotensin-converting enzyme (ACE) inhibitors [136]. Although initial trials are promising, the results of phase III clinical trials are being awaited [136,137,138,139,140]

## 6. Conclusions

Despite great progress in cardiovascular research, CAD remains one of the most common causes of morbidity and mortality worldwide. However, significant inter-collaborative efforts between researchers, clinicians and other related professionals have led to multi-faceted and novel strategies to be developed to treat CAD and its associated conditions. Though some of these strategies have strong evidence supporting their clinical use, some others are still in the experimental stage. Despite only early evidence being available on some of these novel treatment modalities, the results are promising and hold the potential to become alternatives to current treatment options in the future. Since we live in the era of evidence-based medicine, further evidence in the form of clinical trials and long term follow up studies are required before these novel treatment strategies enter into mainstream practice. With sustained continued efforts, the future for CAD therapeutics looks substantially promising.

## Figures and Tables

**Table 1:** Summary of clinical studies for robotic assisted coronary artery bypass grafting.

S. No.	Author’s Name	Results	Additional Comments
1.	Dogan et al. [17]	They reported a patency rate of 100%.	TECAB was performed on hearts arrested intraoperatively.
2.	Kappert et al. [18]	Reduced duration of surgery (down from 280 to 186 minutes); All of them had normal wound healing	TECAB was performed on a beating heart. 3 patients had to undergo re-exploration due to bleeding.
3.	Mohr et al. [19]	Successful procedure in 22 patients (5 of them had to be converted to manual procedure); At discharge, patency was 100% and 95.4% at 3 months; In the TECAB group, success rate was 50%.	TECAB was performed on beating (*n* = 8) and arrested (*n* = 27) heart.

TECAB: Totally endoscopic coronary artery bypass graft; S. No: Serial number.

## Abbreviations

ACE	Angio-tensin-converting enzyme
ARNi	Angiotensin receptor-neprilysin inhibitor
CAD	Coronary artery disease
CABG	Coronary artery bypass graft
HDL	High-density lipoprotein
LCZ696	Combination of sacubitril and valsartan
LVEF	Left ventricular ejection fraction
LDL	Low-density lipoprotein
MI	Acute myocardial infarction
PCI	Percutaneous coronary intervention
PCSK9	Proprotein convertase subtilisin/kexin type 9
TECAB	Totally endoscopic coronary bypass surgery
